# Synthesis, structural studies, and inhibitory potential of selected sulfonamide analogues: insights from in silico and in vitro analyses

**DOI:** 10.17179/excli2024-8118

**Published:** 2025-04-01

**Authors:** Tahira Noor, Daniel C. Schultz, Gustavo Seabra, Yuting Zhai, Kwangcheol Casey Jeong, Saleem Ahmed Bokhari, Fahim Ashraf Qureshi, Abdul Rauf Siddiqi, Chenglong Li

**Affiliations:** 1 Department of Biosciences, COMSATS University Islamabad (CUI), Park Road, Islamabad 45550, Pakistan; 2 Department of Medicinal Chemistry, College of Pharmacy, University of Florida, Gainesville, Florida, USA; 3 Department of Bioinformatics, International Islamic University Islamabad (IIUI), Pakistan; 4 Emerging Pathogens Institute, Department of Animal Sciences, University of Florida, Gainesville, Florida, USA

**Keywords:** antibiotic resistance, antimicrobial resistance, computer-aided drug design, sulfonamide

## Abstract

Antimicrobial resistance is a growing public health threat worldwide, and the current drug development pipeline has thus far been inadequate in addressing this impending crisis. Further research into antibiotic agents, both existing and novel, is therefore paramount for identifying suitable candidates to combat antibiotic-resistant pathogens. Sulfonamides, the first class of synthetic antibiotics, target dihydropteroate synthase (DHPS), a key bacterial enzyme. While this class of antibiotics has historically demonstrated great utility, their use has diminished due to resistance and undesired side effects. In the present study, we synthesized a selection of four sulfonamide analogues (**FQ5**, **FQ6**, **FQ7** and **FQ12**), validated their structures through NMR spectroscopy, and evaluated their inhibitory potential through computational docking and MIC assays against four bacterial strains: *Staphylococcus aureus* ATCC 25923, *Pseudomonas aeruginosa* ATCC 27853, *Escherichia coli* ATCC 35401 and *Bacillus subtilis* ATCC 6633. Each compound exhibited antibacterial activity; **FQ5** demonstrated the most potent activity, with an MIC of 32, 16, 16, and 16 µg/mL against aforementioned strains, respectively. **FQ6**, **FQ7** and **FQ12**, on the other hand, exhibited moderate activity against *P. aeruginosa* and *E. coli *(MIC = 128 µg/mL each) and low activity against *S. aureus* and *B. subtilis *(MIC = 256 µg/mL each). Molecular docking studies indicated that **FQ5** captures multiple hydrogen bonding, ionic, and π-π interactions with key binding pocket residues of DHPS, and **FQ5** also demonstrated superior predicted drug-likeness in *in silico* ADMET studies compared to other compounds. **FQ5** is therefore a favorable starting point for further optimization.

## Introduction

Microbial infections present a significant global health threat. The rise of aggressive, life-threatening infections, coupled with the exponential growth in antibiotic-resistant bacterial strains, has intensified the need for novel antibacterial drugs. Indeed, drug-resistant infections could cause up to 10 million deaths per year by 2050 if no action is taken (O'Neill, 2016[23]). Among the bacterial pathogens listed in the 2024 WHO priority list, antibiotic-resistant strains of *Pseudomonas aeruginosa *(*P. aeruginosa*) and* Staphylococcus aureus* (*S. aureus*) have been identified as high-priority pathogens for which novel treatments are needed (WHO, 2024[37]). Another pathogen whose resistance has become increasingly problematic is *Escherichia coli *(*E. coli*), a versatile microorganism found in an array of locations, including water, soil, and the human gastrointestinal tract, and whose infections are associated with numerous diarrheal illnesses (Mueller and Tainter, 2023[22]). Despite the global spread of antibiotic resistance among common bacterial pathogens and the significant need for novel treatment options, however, the antimicrobial drug development pipeline remains (Levy and Marshall, 2004[17]; Boucher et al., 2009[4]). Therefore, further research into existing and novel antibacterial agents is paramount for overcoming these challenges.

**Figure 1:**
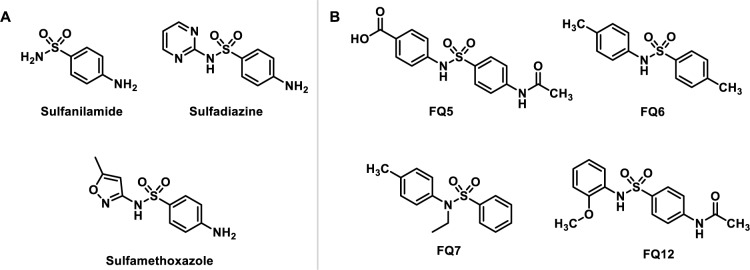
Structures of (A) approved sulfonamide drugs and (B) sulfonamide analogues synthesized in the present study

Sulfonamides, first synthesized in the 1930s, represent the earliest class of synthetic antibiotics and have been widely applied to treat various infections caused by Gram-positive and Gram-negative bacteria (Lemke et al., 2008[16]). These antibiotics function by targeting dihydropteroate synthase (DHPS), a key enzyme in bacterial folate biosynthesis (Roland et al., 1979[28]). DHPS facilitates the condensation of *para*-aminobenzoic acid (*p*ABA) with 6-hydroxymethyl-7,8-dihydropterin-pyrophosphate (DHPPP), producing pyrophosphate and 7,8-dihydropteroate, the latter of which is subsequently converted into dihydrofolate via dihydrofolate synthase (DHFS), then tetrahydrofolate via dihydrofolate reductase (DHFR) (Bertacine Dias et al., 2018[3]). Sulfonamides, such as sulfanilamide, sulfadiazine, and sulfamethoxazole (Figure 1A(Fig. 1)), mimic *p*ABA and competitively inhibit DHPS by binding to its *p*ABA site, disrupting the synthesis of folic acid, a crucial cofactor for bacterial DNA replication, effectively halting bacterial proliferation (Roland et al., 1979[28]; Brown, 1962[5]). Since DHPS is not present in humans, it has been an attractive target for selective antibacterial intervention (Sköld, 2000[33]).

Despite the historical significance of sulfonamides, the severe side effects associated with this class and the significant antimicrobial resistance toward these agents have prompted a shift toward the discovery of alternative antibacterial treatments (Kim et al., 2019[13]; Werth, 2024[36]). Indeed, sulfonamide-derived DHPS inhibitors are notably absent from the recent clinical pipeline (Butler et al., 2023[6]; Melchiorri et al., 2024[20]). A number of institutions and academic labs, however, have remained undeterred in the pursuit of novel sulfonamide antibiotics in an effort to overcome common resistance mechanisms and improve their drug properties (Esfahani et al., 2021[11]; Sabry et al., 2022[29]; Xie et al., 2023[31]; Krátký, 2024[15]). The present work is one such study contributing to the body of knowledge surrounding existing and novel sulfonamides in an effort to better inform the drug development community regarding the properties of compounds within this class. Herein, we describe the use of *in vitro *assays for antibacterial activity assessment and an array of computational tools for evaluating drug-target interactions and drug-like property predictions for a number of synthesized sulfonamide analogues (Figure 1B(Fig. 1)). *In silico *tools have been widely cited as a reliable and a cost-effective and time-efficient means of screening compounds in the drug development pipeline (Yu and MacKerell Jr, 2017[40]). Our computational strategy involved molecular docking studies and *in silico *prediction of Adsorption, Distribution, Metabolism, Excretion, and Toxicity (ADMET) properties of these compounds.

## Materials and Methods

### Chemistry - general information

The synthesis of sulfonamide analogues was carried out using standard techniques under air, unless otherwise specified. Reagents and solvents were purchased from chemical vendors and used without purifycation. Reactions were monitored via thin layer chromatography (TLC). NMR spectra were obtained using a Bruker Avance NEO-600 Spectrometer (^1^H NMR: 600 MHz; ^13^C NMR: 151 MHz). All spectra were visualized using MestReNova 11.0, and all structures shown were drawn using ChemDraw 18.1. The solvent used for obtaining spectral data was DMSO-*d6* (^1^H NMR: 2.50 ppm, ^13^C NMR: 39.52 ppm). All peaks were referenced either to the solvent peak or to TMS (^1^H NMR: 0.00 ppm, ^13^C NMR: 0.00 ppm). All NMR experiments were conducted at room temperature. NMR spectra can be found in the Supplementary Data.

### Chemistry - synthesis of sulfonamide analogues

#### 4-((4-Acetamidophenyl)sulfonamido)benzoic acid (FQ5):

*Para*-acetylaminobenzenesulfonyl chloride (3 g, 0.01 mol, 1 eq.) was dissolved in 20 mL diethyl ether. *Para*-amino benzoic acid (3g, 0.02 mol, 2 eq.) was added slowly to the acetanilide solution with stirring while maintaining the pH of the reaction mixture between pH 7-8 using 3 % Na_2_CO_3_ solution over a period of three hours. Resulting product crystals were collected, washed with distilled water, and recrystallized using 95 % denatured ethanol to afford the title compound (93 % yield).

**^1^H NMR: **(600 MHz, DMSO-*d_6_*) δ 12.71 (s, 1H), 10.69 (s, 1H), 10.31 (s, 1H), 7.82 - 7.77 (m, 2H), 7.77 - 7.72 (m, 2H), 7.72 - 7.69 (m, 2H), 7.20 - 7.15 (m, 2H), 2.05 (s, 3H). **^13^C NMR:** (151 MHz, DMSO-*d_6_*) δ 169.1, 166.8, 143.4, 142.1, 132.7, 130.7, 128.1, 125.5, 118.7, 118.1, 24.1. ^1^H and ^13^C NMR match literature (Pastor-Navarro et al., 2004[25]) CAS: 72236-24-9.


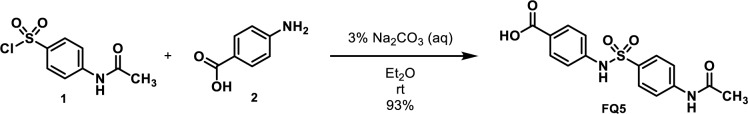


#### 4-Methyl-N-(p-tolyl)benzenesulfonamide (FQ6): 

Tosyl chloride was added dropwise to an 0.05 M solution of *p*-toluidine (5.35 g, 0.05 mol) in distilled H_2_O (1 L) while maintaining the pH between 7-8 using a 3 % Na_2_CO_3_ solution. The reaction mixture was stirred for at least two hours, then the product was washed with distilled water and re-crystallized using 95 % denatured ethanol to afford the title compound (92 % yield).

**^1^H NMR: **(600 MHz, DMSO-*d_6_*) δ 10.02 (s, 1H), 7.62 - 7.57 (m, 2H), 7.35 - 7.29 (m, 2H), 7.03 - 6.98 (m, 2H), 6.98 - 6.93 (m, 2H), 2.32 (s, 3H), 2.17 (s, 3H). **^13^C NMR**: (151 MHz, DMSO-*d_6_*) δ 143.1, 136.7, 135.1, 133.2, 129.6, 129.5, 126.7, 120.5, 20.9, 20.3. ^1^H and ^13^C NMR match literature (Zhang et al., 2015[41]) CAS: 599-86-0.


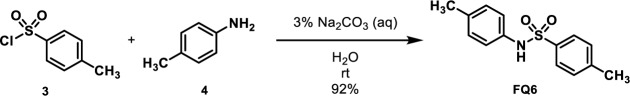


#### N-ethyl-N-(p-tolyl)benzenesulfonamide (FQ7): 

Benzenesulfonyl chloride (9.8 g, 55 mmol, 1.1 eq) was added slowly to an 0.05 M solution of *p*-toluidine (5.35 g, 0.05 mol, 1.0 eq) in distilled water over a period to two hours while maintaining the pH between 7-8 using a 3 % Na_2_CO_3_ solution. Upon completion, a 1 M ethyl iodide solution (prepared by adding 0.5 mol ethyl iodide to 0.5 L diethyl ether) was added dropwise while stirring over a period of two hours. The resulting product crystals were collected, washed with distilled water, and recrystallized using 95 % denatured ethanol to afford the title compound (85 % yield).

**^1^H NMR: **(600 MHz, DMSO-*d_6_*) δ 7.72 - 7.65 (m, 1H), 7.62 - 7.53 (m, 4H), 7.18 - 7.12 (m, 2H), 6.92 - 6.86 (m, 2H), 3.56 (q, *J* = 7.1 Hz, 2H), 2.29 (s, 3H), 0.95 (t, *J* = 7.1 Hz, 3H). **^13^C NMR**: (151 MHz, DMSO-*d_6_*) δ 137.9, 137.3, 135.7, 133.0, 129.5, 129.2, 128.3, 127.2, 45.1, 20.6, 13.8. This compound has been reported (Debnath and Mondal, 2018[10]; Wagner, 1933[35]) but no NMR spectra have been published in DMSO-*d_6_* for comparison. CAS: 1379613-40-7.


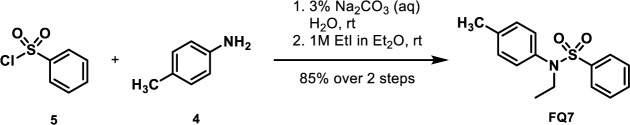


#### N-(4-(N-(2-methoxyphenyl)sulfamoyl)phenyl)acetamide (FQ12):

Briefly, *para*-acetylaminobenzenesulfonyl chloride (0.52 g, 2.2 mmol, 1.0 eq) was added slowly, with stirring, to a mixture of *o*-anisidine (0.27 g, 2.2 mmol, 1.0 eq) in diethyl ether (5 mL) over a period of two hours at room temperature while maintaining the pH between 7-8 using 3 % Na_2_CO_3_ solution. The resulting product crystals were collected, washed with double distilled water, and recrystallized using 95 % denatured ethanol to afford the title compound (90 % yield).

**^1^H NMR: **(600 MHz, DMSO-*d_6_*) δ 10.26 (s, 1H), 9.28 (s, 1H), 7.70 - 7.64 (m, 2H), 7.64 - 7.59 (m, 2H), 7.19 (dd, *J* = 7.9, 1.7 Hz, 1H), 7.09 (td, *J* = 7.8, 1.6 Hz, 1H), 6.92 - 6.87 (m, 1H), 6.85 (td, *J* = 7.6, 1.3 Hz, 1H), 3.51 (s, 3H), 2.06 (s, 3H). **^13^C NMR:** (151 MHz, DMSO-*d_6_*) δ 169.0, 152.1, 142.8, 134.1, 127.9, 126.4, 125.5, 124.7, 120.4, 118.2, 111.8, 55.5, 24.1. ^1^H and ^13^C NMR align with literature (Kowalik et al., 2021[14]) CAS: 19838-01-8.


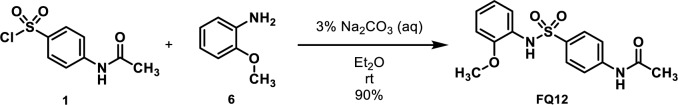


## Computational Experiments

### Target protein preparation 

The crystal structure of a ternary complex of *Escherichia coli* dihydropteroate synthase complexed with sulfanilamide and DHPPP, obtained by X-Ray diffraction at a 2.0 Å resolution, was retrieved from the RCSB Protein Data Bank (PDB: 1AJ0) (Achari et al., 1997[1]). The protein was then prepared for docking using Maestro Schrödinger (version 13.2) (Schrödinger, LLC, 2023[31]). Missing residues and loop segments close to the active site were added using Prime, and bound ligands (DHPPP and sulfanilamide) and sulfates were removed. Hydrogen atoms were added after deleting any original ones, and proper bond orders were assigned. In addition, water molecules were removed except for WAT 308, which coordinates with the Mg^2+^ ion. PROPKA was used to sample hydrogen bonds while adjusting the orientations of the water molecules in the active site at pH 7.0 (Olsson et al., 2011[24]). After that, the geometry of the protein-ligand complex was refined using OPLS4 force field restrained minimization with convergence of heavy atoms to an RMSD of 0.3 Å (Harder et al., 2016[12]).

### Docking

Docking was performed with QuickVina 2.1 (Alhossary et al., 2015[2]). The docking grid box was centered on the sulfanilamide binding site, and 9 poses were generated for each compound. Redocking of sulfanilamide was carried out to test the adopted protocol. After docking, the poses obtained were rescored with DeepAtom, a Deep Learning model for estimating binding affinities (Rezaei et al., 2022[27]).

### Minimum Inhibitory Concentration assay 

Minimum Inhibitory Concentration (MIC) assays were performed using four standard strains of Gram-positive and Gram-negative bacteria, including *Escherichia coli* ATCC 35401, *Pseudomonas aeruginosa* ATCC 27853, *Staphylococcus aureus* ATCC 25923, and *Bacillus subtilis* ATCC 6633. The MICs were determined using a micro-broth dilution method that followed the Clinical and Laboratory Standards Institute (CLSI) guidelines (CLSI, 2018[9]). The potential antibiotic compounds were solubilized in dimethyl sulfoxide and tested at concentrations that spanned the doubling dilution range from 256 μg/mL to 0 μg/mL. Mueller Hinton Broth was used for diluting the antimicrobial stocks. The overnight bacteria culture was diluted into 5 mL of fresh Tryptic Soy Broth and grew to an optical density (OD600) of one. Then, the bacteria culture was diluted 100 times, two microliters of which was added to the well with 198 μL antimicrobial diluents in the 96-well plates. The MICs of bacteria were estimated after 24-hour growth with shaking (200 rpm) at 37 ℃ (Table 1(Tab. 1)).

### In silico prediction of molecular properties and drug-likeness

Molecular properties, drug-likeness, and ADMET data for the synthesized sulfonamide derivatives were predicted using ADMET Predictor® (Simulations Plus), Schrödinger's QikProp (Schrödinger, LLC[31]), Molsoft (MolSoft LLC[21]), and PkCSM (Pires et al., 2015[26]).

## Results and Discussion

Three sulfonamide analogues were synthesized via the addition of an arylsulfonyl chloride to the appropriate aniline, affording the desired compounds (**FQ5**, **FQ6**, and **FQ12**) in 90-93 % yield, with a fourth analogue, **FQ7**, synthesized in 85 % overall yield via the sequential addition of benzenesulfonyl chloride and ethyl iodide to *p*-toluidine (Figure 1B(Fig. 1)). Each of these compounds features two phenyl rings linked by a sulfonamide moiety, with various functional groups decorating the aromatic rings, including acetamide, methoxy, methyl, and carboxylic acid groups. In order to provide a baseline for the evaluation of these compounds, these were assessed for their antibacterial activity in a Minimum Inhibitory Concentration (MIC) assay against four model bacterial strains, including *P. aeruginosa *(ATCC 25923),* S. aureus *(ATCC 27853)*, E. coli *(ATCC 35401), and *Bacillus subtilis *(*B. subtilis; *ATCC 6633) (Table 1(Tab. 1), Supplementary Figures 6-9).

Among these four compounds, **FQ5 **exhibited the best antibacterial activity against the tested strains of bacteria, demonstrating an MIC of 32 µg/mL against *S. aureus *and 16 µg/mL against *P. aeruginosa*, *E. coli*, and *B. subtilis*. The remaining compounds, **FQ6**, **FQ7**, and **FQ12**, however, exhibited many-fold weaker MICs against all tested bacteria compared to **FQ5**. 

**Figure 2:**
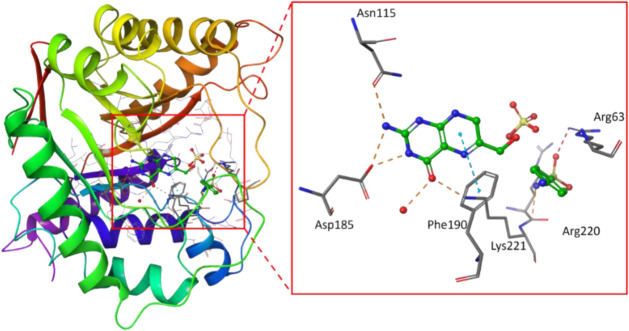
Structure of ecDHPS from PDBID:1AJ0. The active site is highlighted on the right, depicting the bound configuration of DHPPP and sulfanilamide. Hydrogen bonds are depicted with red-orange dashes, and π-π interactions are depicted with blue dashes.

**Figure 3:**
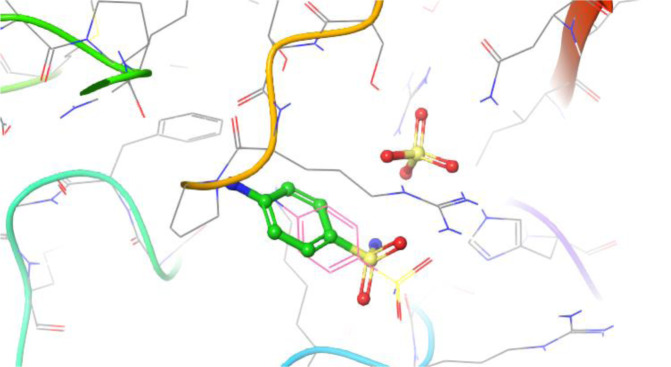
Re-docking of sulfanilamide to the DHPS active site (ball-and-stick model) for comparison to its crystal structure pose (line model)

To rationalize this differential in activity, we docked each of these compounds against the target enzyme, DHPS, using QuickVina 2.1 (Alhossary et al., 2015[2]). The crystal structure of sulfanilamide and DHPPP bound to *E. coli *DHPS was solved in 1997 (PDBID 1AJ0) and served as the basis for this pursuit (Achari et al., 1997[1]). Within this crystal structure, it is evident that sulfanilamide captures multiple hydrogen bonding interactions with Arg63 and Arg220 (Figure 2(Fig. 2)). Prior to docking the sulfonamide compounds synthesized in the present study, sulfanilamide was re-docked in order to validate the docking protocol, and the docked pose of sulfanilamide was found to align with the crystal structure pose within an RMSD of 2.5 Å (Figure 3(Fig. 3)).

**Figure 4:**
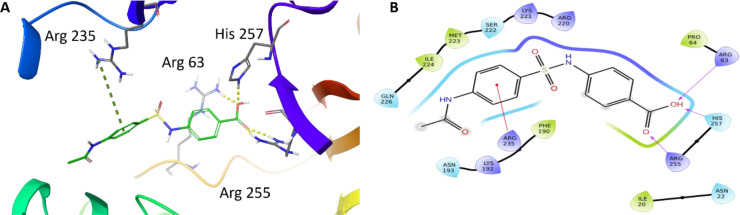
(A) Binding mode of compound FQ5 docked to DHPS binding pocket. Key DHPS residues are indicated. Yellow dashed lines represent hydrogen bonds and ionic interactions. Green dashed lines shows cation-π interactions. (B) Interaction diagram showing key FQ5 interactions with DHPS residues. Residues are represented in three letter code with their position. Purple arrows represent hydrogen bonds and ionic interactions. Red lines shows the cation-π interactions.

**Figure 5:**
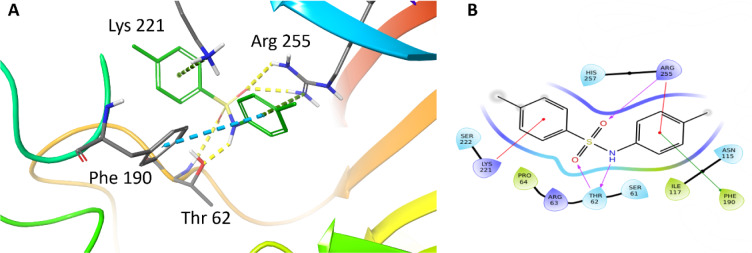
(A) Binding mode of FQ6 docked to the DHPS binding pocket. DHPS residues exhibiting important interactions are indicated. Yellow dashed lines represent hydrogen bonds. Green dashed lines show cation-π interactions. Blue dashed lines show π-π interactions. (B) Interaction diagram showing interactions between FQ6 and DHPS. Residues are represented in three letter code with their position. Arrows in purple represent hydrogen bonds with donor at the base and acceptor at the arrowhead. Red lines show the cation-π interactions, and green lines show π-π interactions.

**Figure 6:**
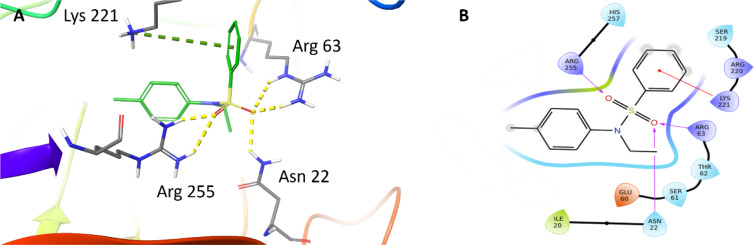
(A) Binding mode of FQ7 docked to the DHPS binding pocket. Key residues are indicated. Yellow dashed lines represent hydrogen bonds. (B) Interaction diagram showing FQ7 interactions with DHPS. Residues are represented in three letter code with their position. Arrows in purple represent hydrogen bonds with donor at the base and acceptor at the arrowhead, and red lines shows the cation-π interactions.

**Figure 7:**
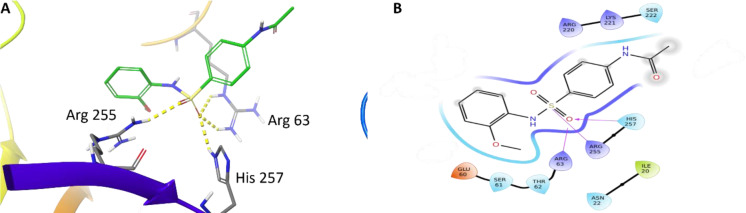
(A) Binding mode of FQ12 docked to the DHPS binding pocket. Key residues are indicated. Yellow dotted lines represent hydrogen bonds. (B) Interaction diagram showing interactions between FQ12 and DHPS. Residues are represented in three letter code with their position. Arrows in purple represent hydrogen bonds with donor at the base and acceptor at the arrowhead.

With our docking method validated, we then docked each of the presented sulfonamide analogues to the active site of DHPS (Figures 4-7(Fig. 4)(Fig. 5)(Fig. 6)(Fig. 7)) and re-scored their best docking poses using DeepAtom (Table 2(Tab. 2)) (Rezaei et al., 2022[27]). **FQ5 **captures multiple hydrogen bonds and ionic interactions between its carboxylate and Arg63, His257, and Arg255, as well as cation-π interactions between one of its phenyl rings and Arg235 (Figure 4(Fig. 4)). **FQ6 **displays similar interactions, including π-π interactions with Arg255 and Phe190, and it notably exhibits dual hydrogen bonding between its sulfonamide and Thr62 (Figure 5(Fig. 5)). Tertiary sulfonamide **FQ7**, however, exhibits multiple hydrogen bonding interactions between its sulfonamide oxygens and Asn22, Arg63, and Arg255, as well as a cation-π interaction with Lys221 (Figure 6(Fig. 6)). Finally, **FQ12 **exhibits solely hydrogen bonding interactions within the DHPS *p*ABA binding site, capturing interactions with Arg63, Arg255, and His257.

Based on the proposed bound poses for these compounds, it is evident that the superior antibacterial activity of **FQ5** is driven by its carboxylate group since the functional groups decorating the remaining analogues are not projected to capture any substantial interactions with DHPS beyond simple hydrophobic interactions. 

Given that a significant portion of clinical trial failures are due to poor drug-like properties (10 to 15 % of failures) or toxicity (30 % of failures), the use of computational tools to predict these properties has become increasingly valued to reduce the risk of late-stage drug candidate failure (Sun et al., 2022[34]; Wu et al., 2020[38]). Therefore, we applied a variety of computational tools, including ADMET Predictor® (Simulations Plus), Schrödinger's QikProp (Schrödinger, LLC[31]), Molsoft (MolSoft LLC[21]), and PkCSM (Pires et al., 2015[26]) to predict the drug-likeness, physicochemical properties, and ADMET properties of these sulfonamide analogues (Tables 3(Tab. 3) and 4(Tab. 4)).

To begin, we evaluated the drug-likeness of these compounds on the basis of Lipinski's Rule of 5, and each of these were predicted to fall within the acceptable ranges for these parameters (Table 3(Tab. 3)) (Lipinski et al., 1997[19]). Another important property to consider is molecular polar surface area (MolPSA), which has been used for nearly three decades as a means of predicting both intestinal absorption and blood-brain barrier penetration (Clark, 1999[7][8]). Three of the four compounds were calculated to have a polar surface area of less than 90 Å^2^, though **FQ12** was only slightly above this cutoff. When taking Lipinski's parameters and MolPSA into account, only **FQ5** and sulfadiazine, which was used as a control during these *in silico *studies, were flagged as drug-like compounds.

These sulfonamide analogues were then subjected to *in silico *assessment of a variety of ADMET properties (Table 4(Tab. 4) and Supplementary Table 1). All were predicted to have few metabolites and lack human ether-a-go-go related gene (hERG) potassium channel liabilities. Interestingly, both sulfadiazine and **FQ5** were predicted to have undesired human intestinal absorption. While no compounds were predicted to act as inhibitors of P-glycoprotein (P-gP), a key transporter which plays a significant role in removing xenobiotics from the central nervous system (Lin and Yamazaki, 2003[18]), **FQ5 **and **FQ12** are both predicted to act as P-gP substrates, suggesting poor brain penetrance.

With respect to predicted pharmacokinetic parameters, **FQ5** and **FQ12** are predicted to have a high C_max_ and moderate half-lives, while **FQ6** and **FQ7 **are predicted to have a lower C_max_ and much longer half-lives. It is noted, however, that sulfadiazine is predicted to have an *in vivo *half-life of 1.18 hours, which is significantly lower than its published half-life (Scholar, 2007[30]). Finally, all compounds were predicted to have a high fraction absorbed ( %Fa) and high oral bioavailability ( %Fb). Overall, **FQ5**, the most potent of the compounds synthesized in the present study, is predicted to have favorable ADMET properties for further study and analogue development.

## Conclusion

The synthesis, *in vitro *assessment, and *in silico* evaluation of a selection of sulfonamide antibiotic analogues targeting DHPS, a key bacterial enzyme, is described. Among the tested compounds, **FQ5** exhibited the best potency, with an MIC of 32 µg/mL against *S. aureus *and 16 µg/mL against *P. aeruginosa*, *E. coli*, and *B. subtilis*. Furthermore, docking studies indicate that this compound likely captures multiple ionic, hydrogen bonding, and π-π interactions with key residues of DHPS, providing a strong rationale for its superior activity compared to the other tested sulfonamides. Finally, thorough *in silico *prediction of its ADMET properties provided favorable results, suggesting this compound could be a suitable starting point for further sulfonamide analogue development.

## Notes

Abdul Rauf Siddiqi (Department of Biosciences, COMSATS University Islamabad (CUI), Park Road, Islamabad 45550, Pakistan) and Chenglong Li equally contributed as corresponding author.

## Declaration

### Author contributions

ARS conceived and designed the study and experiments. TN performed the majority of the experiments, analyzed the data. DCS, GS, YZ, and KCJ assisted with experiments and manuscript writing. FAQ and ARS designed and synthesized the sulfonamide analogues. SAB, ARS, and CL revised and improved the paper. ARS and CL supervised the study.

### Conflict of interest

The authors declare no conflict of interest.

### Acknowledgments

The authors thank the Department of Medicinal Chemistry, College of Pharmacy, University of Florida (Gainesville, Florida, USA), for accommodating this research and other members of the Li lab for their overarching support and encouragement. The authors are also thankful for the support and resources of COMSATS University Islamabad and FDP Grant # 17-5/FBSI-002/HEC/ Sch-FDP/2018 of HEC, Pakistan, which provided financial support for Tahira Noor to undertake a one-year secondment under the supervision of Dr. Chenglong Li at the University of Florida. The authors also extend their gratitude to the International Islamic University Islamabad (IIUI) for granting study leave, enabling the completion of this research.

## Supplementary Material

Supplementary data

## Figures and Tables

**Table 1:**
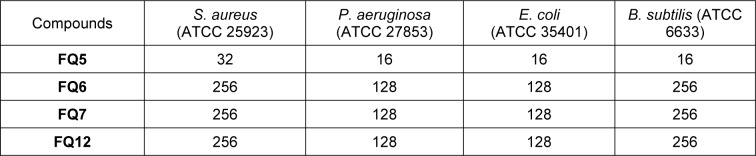
Minimum Inhibitory Concentration (MIC), in µg/mL, of sulfonamide analogues against selected bacterial strains

**Table 2:**
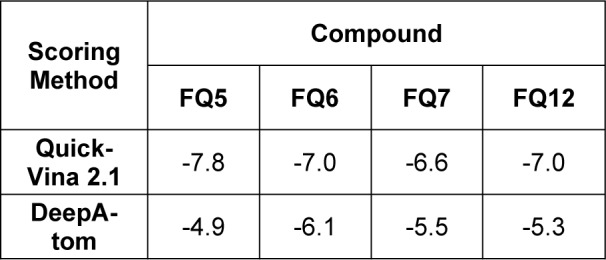
Sulfonamide analogue docking scores (QuickVina 2.1) and DeepAtom scores

**Table 3:**
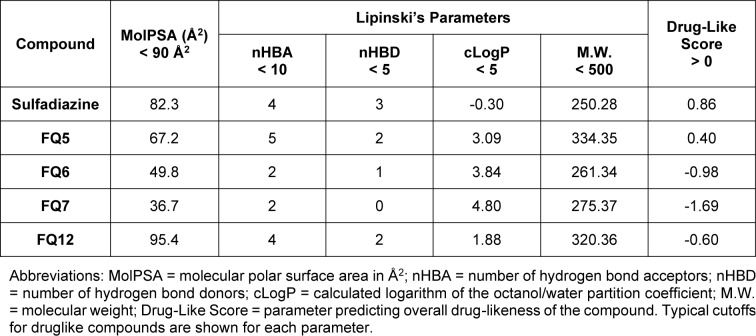
Predicted drug-likeness and molecular properties

**Table 4:**
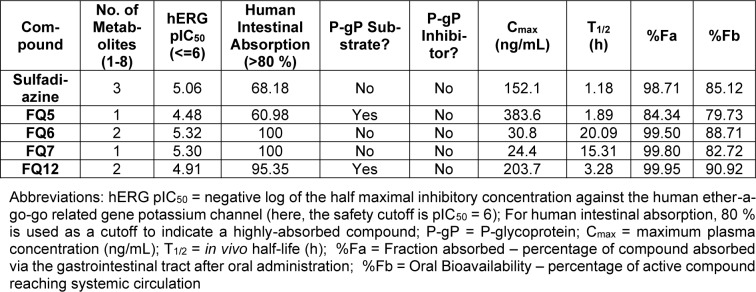
Predicted ADMET properties
