# Supplementary material for: Synthesis, structural studies, and inhibitory potential of selected sulfonamide analogues: insights from in silico and in vitro analyses
Source: EXCLI J. 2025 Apr 1;24:527–38. doi: 10.17179/excli2024-8118 (PMC12078777; doi:10.17179/excli2024-8118)
Supplement: Supplementary data [file EXCLI-24-527-s-001.pdf]

EXCLI Journal 2025;24:527-538 – ISSN 1611-2156
Received: December 17, 2024, accepted: February 25, 2025, published: April 01, 2025

Supplementary data to:

Original article:

SYNTHESIS, STRUCTURAL STUDIES, AND INHIBITORY POTENTIAL OF SELECTED SULFONAMIDE ANALOGUES: INSIGHTS FROM *IN SILICO* AND *IN VITRO* ANALYSES

Tahira Noor[1,2,3], Daniel C. Schultz[2], Gustavo Seabra[2], Yuting Zhai[4], Kwangcheol Casey Jeong[4], Saleem Ahmed Bokhari[1], Fahim Ashraf Qureshi[1], Abdul Rauf Siddiqi [1]*, Chenglong Li[,2]**

[1] Department of Biosciences, COMSATS University Islamabad (CUI), Park Road, Islamabad 45550, Pakistan
[2] Department of Medicinal Chemistry, College of Pharmacy, University of Florida, Gainesville, Florida, USA
[3] Department of Bioinformatics, International Islamic University Islamabad (IIUI), Pakistan
[4] Emerging Pathogens Institute, Department of Animal Sciences, University of Florida, Gainesville, Florida, USA

* Shared corresponding authorship

** **Corresponding author:** Chenglong Li, Department of Medicinal Chemistry, College of Pharmacy, University of Florida, Gainesville, Florida, USA. E-mail: lic@ufl.edu

https://dx.doi.org/10.17179/excli2024-8118

This is an Open Access article distributed under the terms of the Creative Commons Attribution License (http://creativecommons.org/licenses/by/4.0/).

EXCLI Journal 2025;24:527-538 – ISSN 1611-2156
Received: December 17, 2024, accepted: February 25, 2025, published: April 01, 2025

Supplementary Information Contents .... Page

Supplementary Figure 1: ^{1}H and ^{13}C NMR (DMSO-*d6*) spectra for compound FQ5 .... S3
Supplementary Figure 2: ^{1}H and ^{13}C NMR (DMSO-*d6*) spectra for compound FQ6. .... S4
Supplementary Figure 3: ^{1}H and ^{13}C NMR (DMSO-*d6*) spectra for compound FQ7 .... S5
Supplementary Figure 4: HSQC and HMBC spectra (DMSO-*d6*) for compound FQ7 with zoomed- in region and key correlations highlighted S6
Supplementary Figure 5: ^{1}H and ^{13}C NMR (DMSO-*d6*) spectra for compound FQ12 .... S7
Supplementary Figure 6: Minimum Inhibitory Concentration (MIC), in µg/mL, of FQ5 against selected bacterial strains S8
Supplementary Figure 7: Minimum Inhibitory Concentration (MIC), in µg/mL, of FQ6 against selected bacterial strains S8
Supplementary Figure 8: Minimum Inhibitory Concentration (MIC), in µg/mL, of FQ7 against selected bacterial strains S9
Supplementary Figure 9: Minimum Inhibitory Concentration (MIC), in µg/mL, of FQ12 against selected bacterial strains S9
Supplementary Table 1: Additional Predicted ADMET Properties S10

EXCLI Journal 2025;24:527-538 – ISSN 1611-2156
Received: December 17, 2024, accepted: February 25, 2025, published: April 01, 2025

FQ5

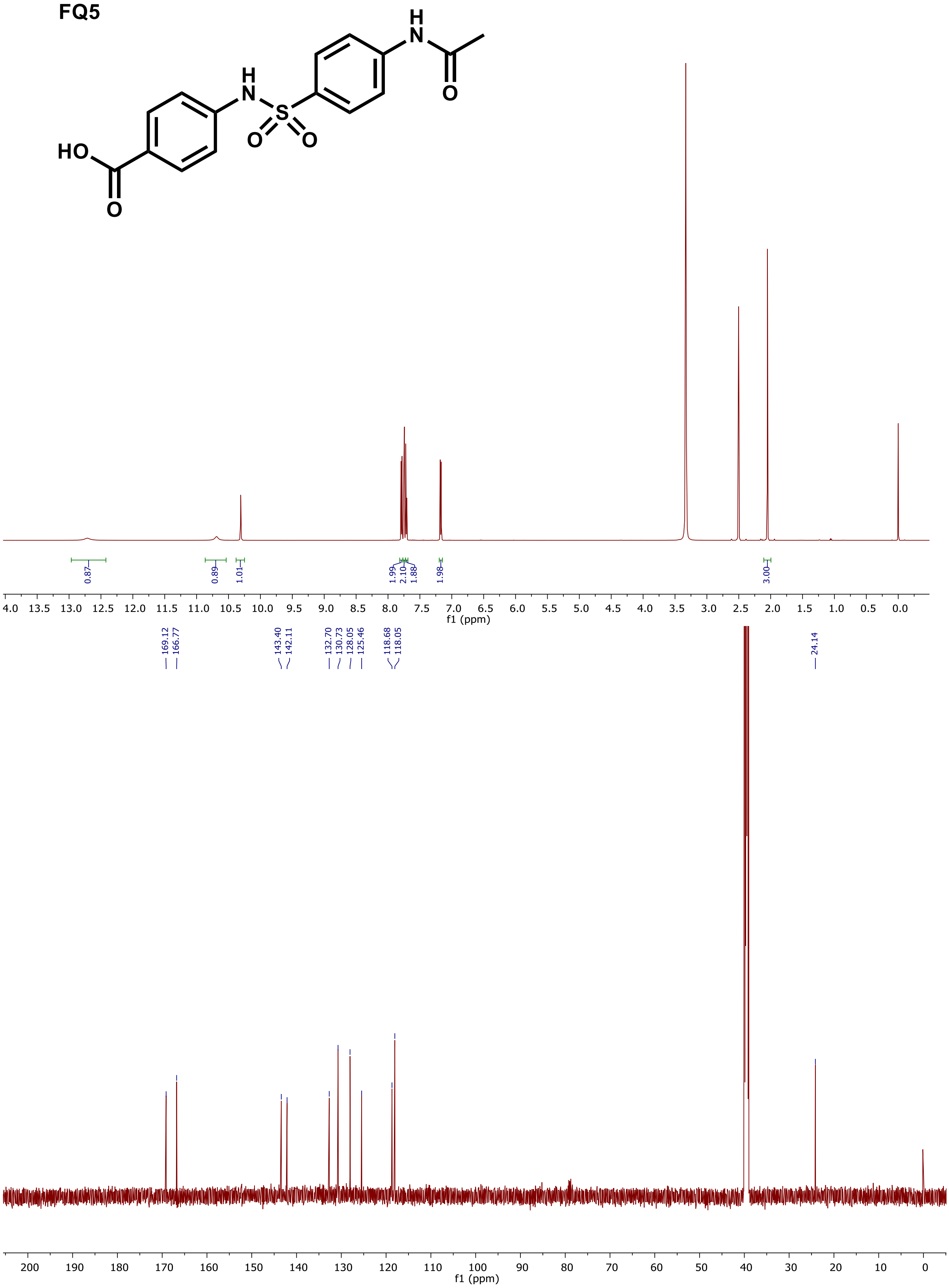

Supplementary Figure 1: ^{1}H and ^{13}C NMR (DMSO-*d6*) spectra for compound FQ5

EXCLI Journal 2025;24:527-538 – ISSN 1611-2156
Received: December 17, 2024, accepted: February 25, 2025, published: April 01, 2025

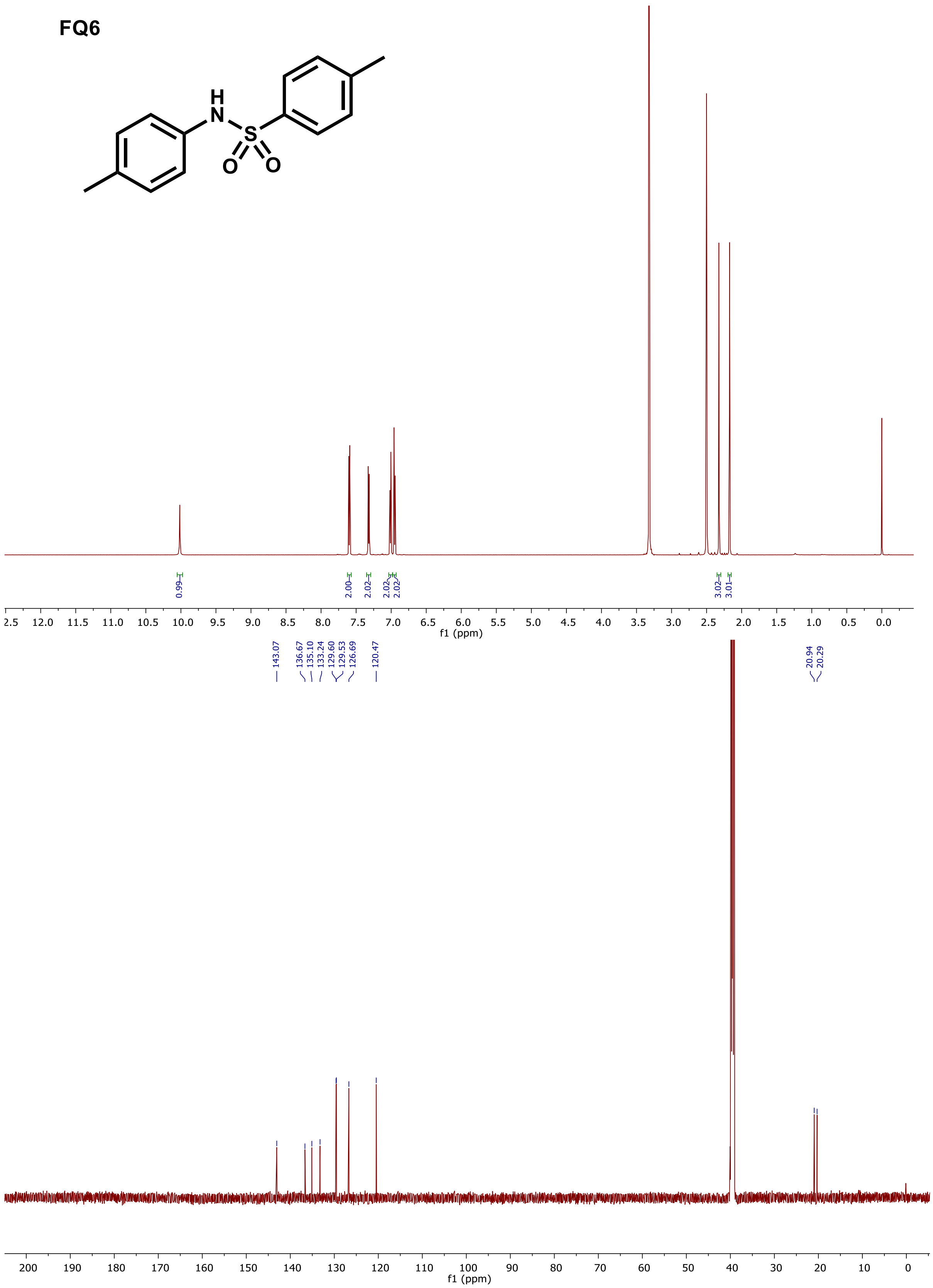

Supplementary Figure 2: ^{1}H and ^{13}C NMR (DMSO-*d6*) spectra for compound FQ6

EXCLI Journal 2025;24:527-538 – ISSN 1611-2156
Received: December 17, 2024, accepted: February 25, 2025, published: April 01, 2025

FQ7

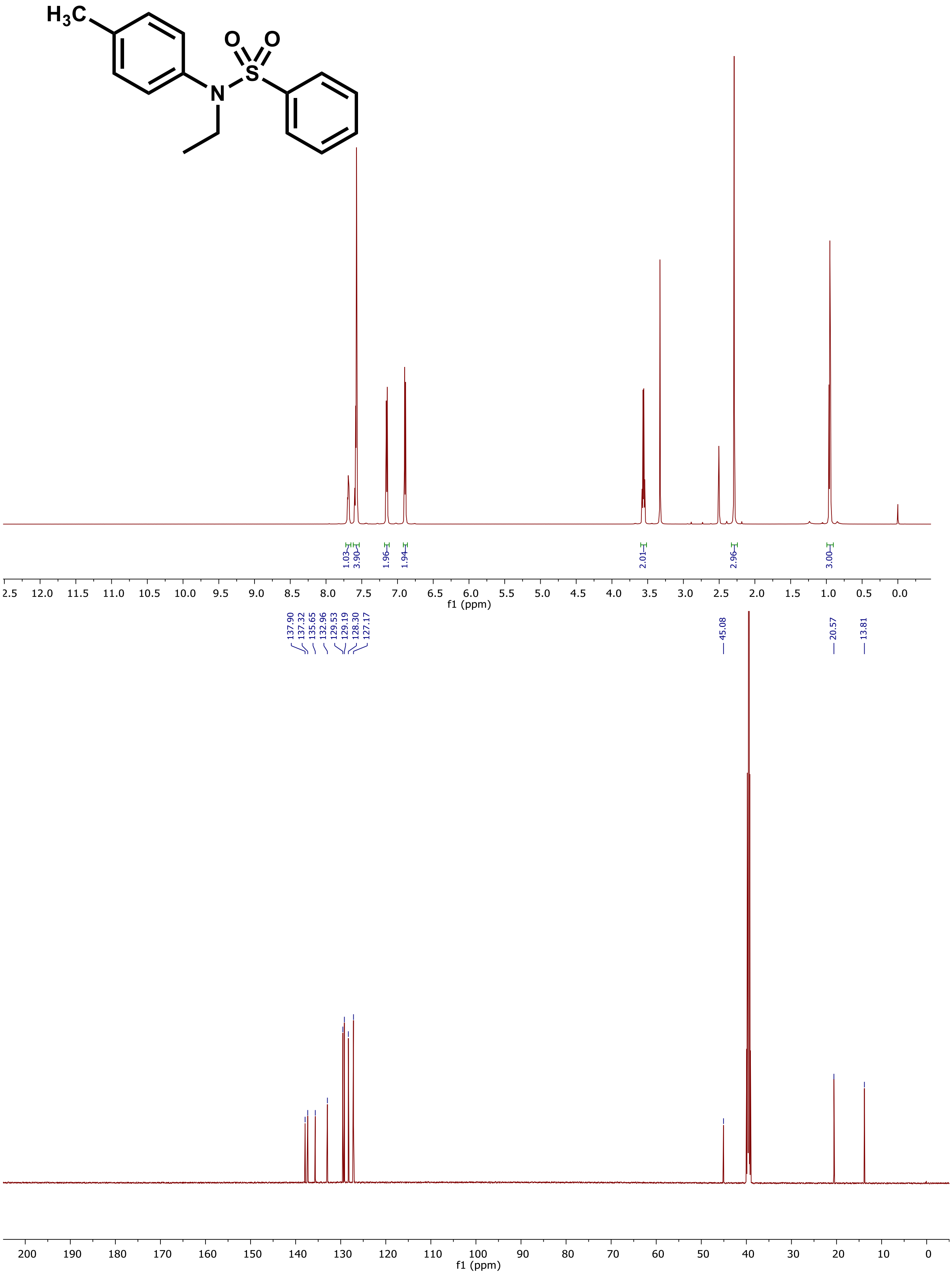

Supplementary Figure 3: ^{1}H and ^{13}C NMR (DMSO-*d6*) spectra for compound FQ7

EXCLI Journal 2025;24:527-538 – ISSN 1611-2156
Received: December 17, 2024, accepted: February 25, 2025, published: April 01, 2025

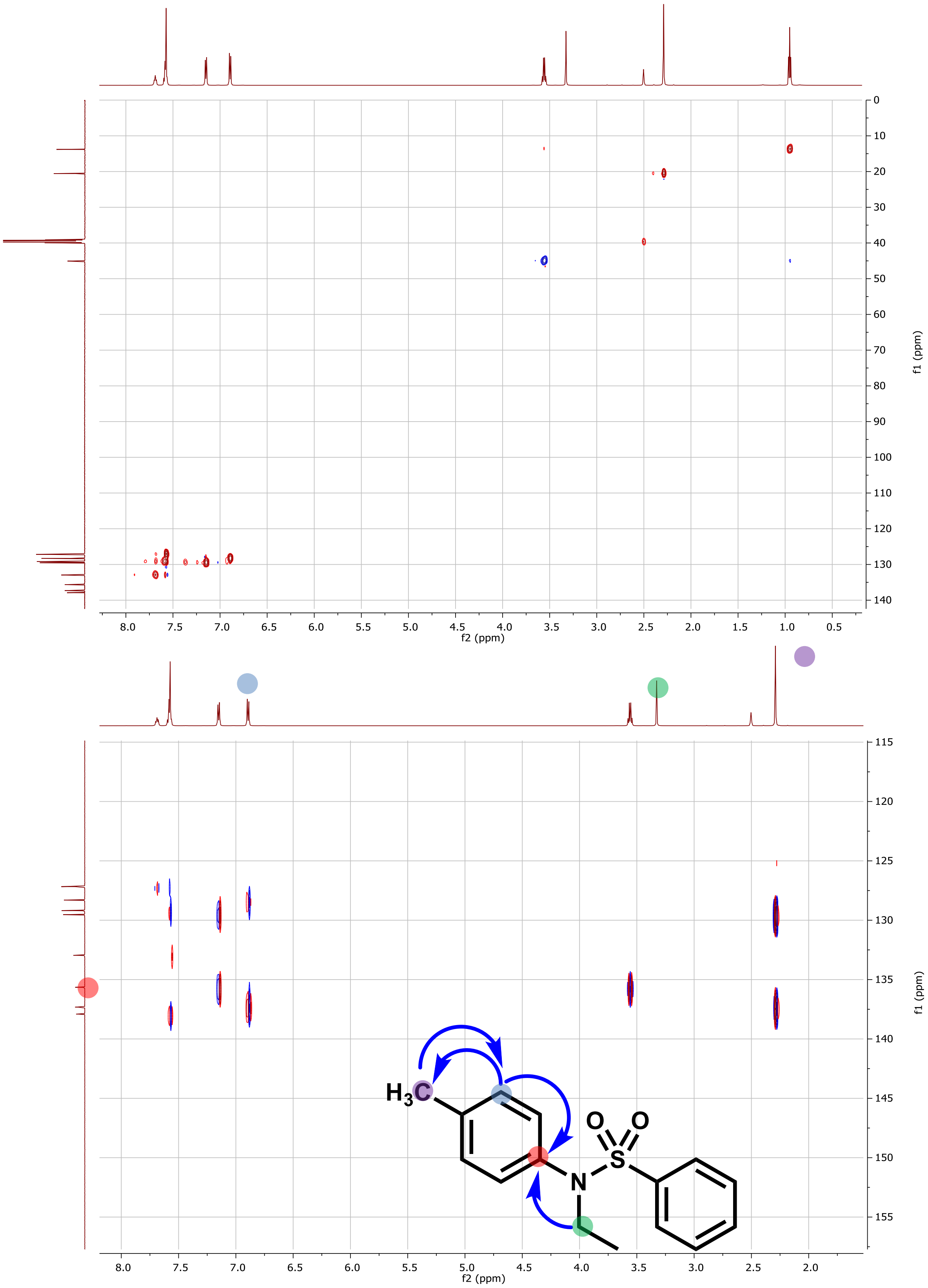

Supplementary Figure 4: HSQC and HMBC spectra (DMSO-*d6*) for compound FQ7, with zoomed- in region and key correlations highlighted

EXCLI Journal 2025;24:527-538 – ISSN 1611-2156
Received: December 17, 2024, accepted: February 25, 2025, published: April 01, 2025

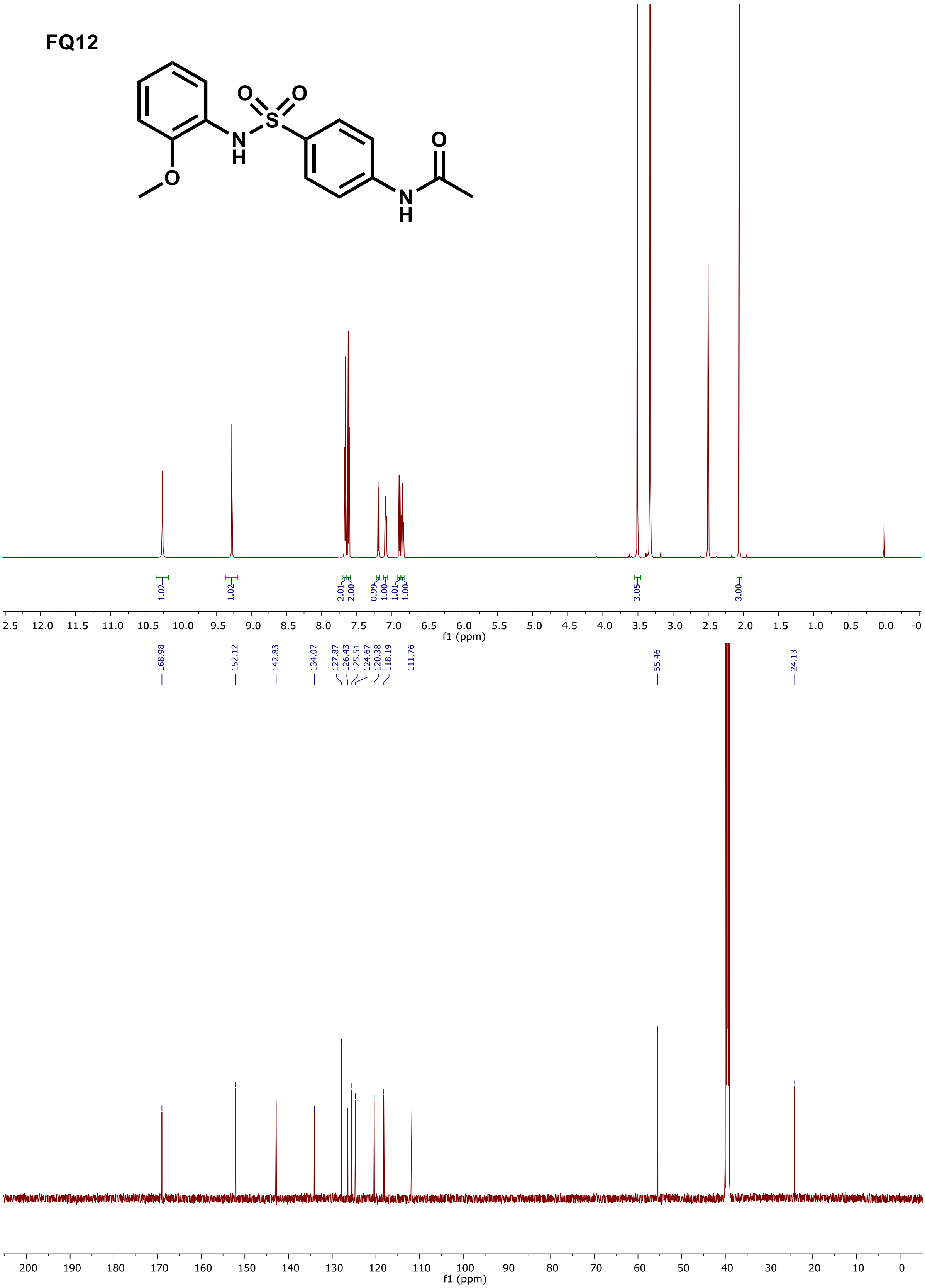

Supplementary Figure 5: ^{1}H and ^{13}C NMR (DMSO-*d6*) spectra for compound FQ12

Received: December 17, 2024, accepted: February 25, 2025, published: April 01, 2025

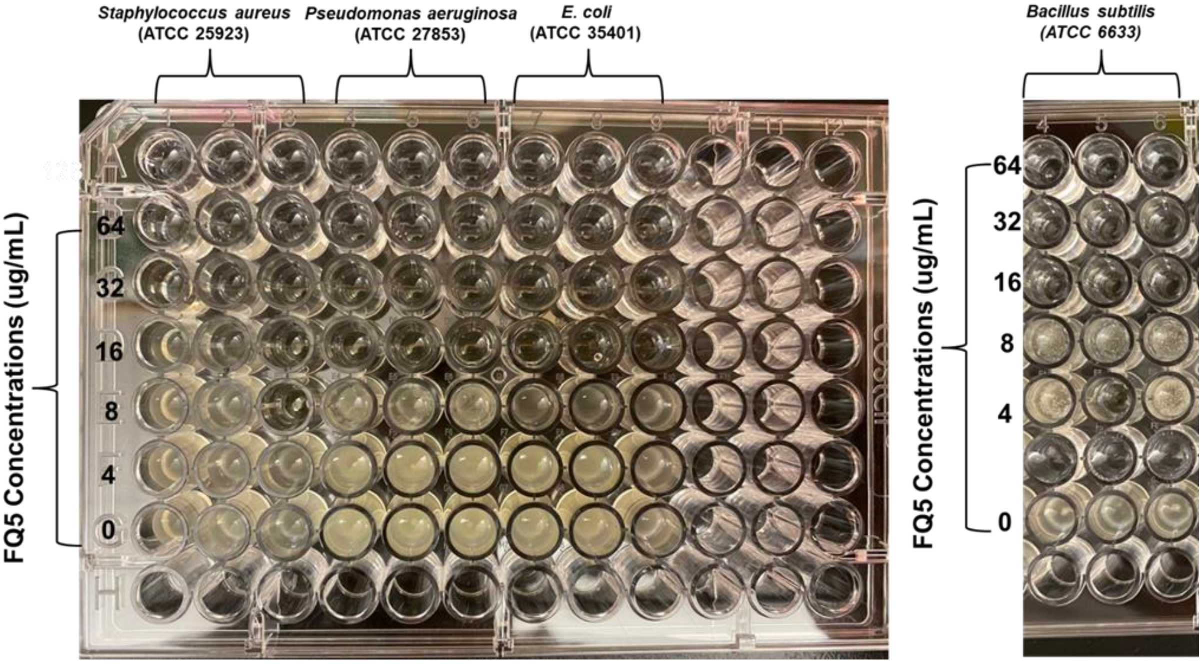

Supplementary Figure 6: Minimum Inhibitory Concentration (MIC), in µg/mL, of FQ5 against selected bacterial strains

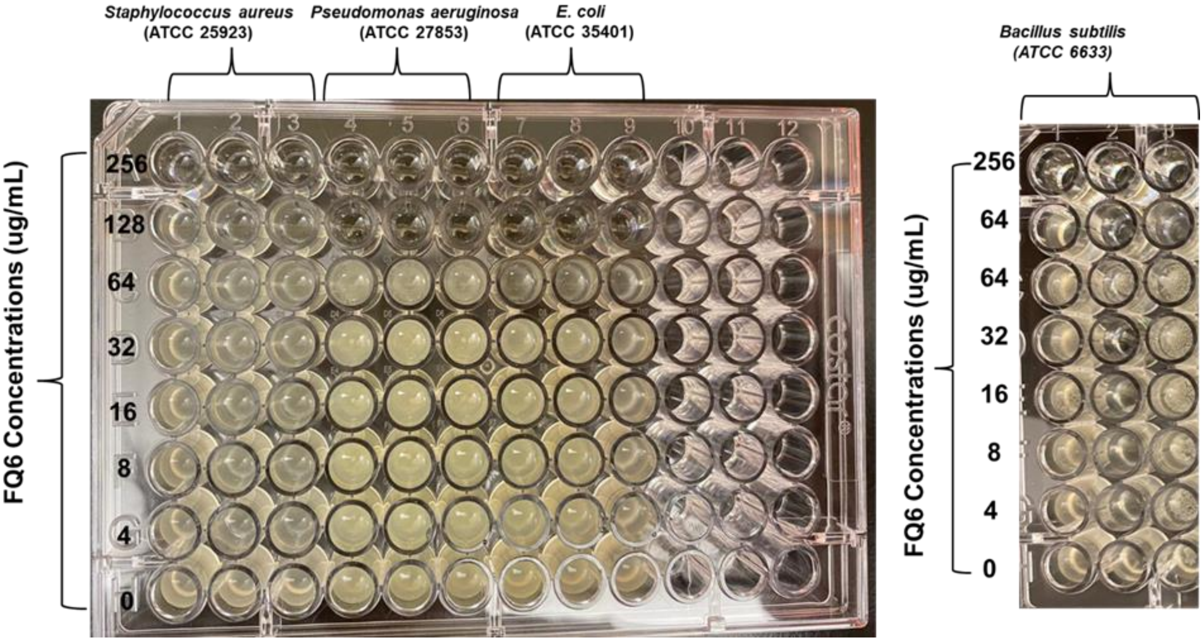

Supplementary Figure 7: Minimum Inhibitory Concentration (MIC), in µg/mL, of FQ6 against selected bacterial strains

EXCLI Journal 2025;24:527-538 – ISSN 1611-2156
Received: December 17, 2024, accepted: February 25, 2025, published: April 01, 2025

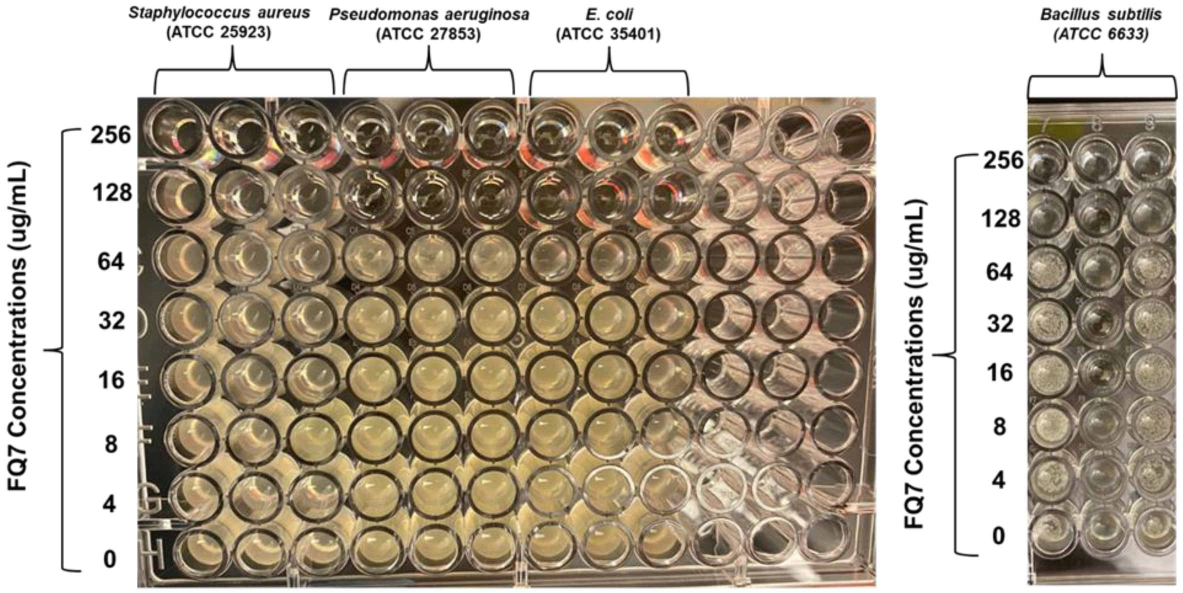

Supplementary Figure 8: Minimum Inhibitory Concentration (MIC), in µg/mL, of FQ7 against selected bacterial strains

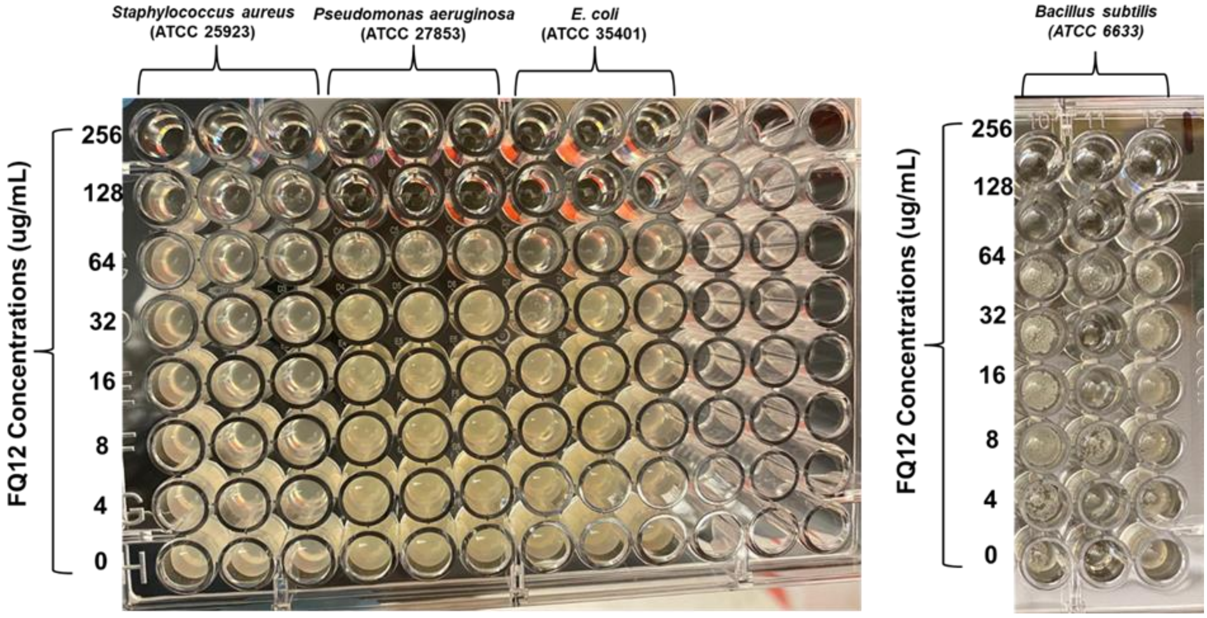

Supplementary Figure 9: Minimum Inhibitory Concentration (MIC), in µg/mL, of FQ12 against selected bacterial strains

EXCLI Journal 2025;24:527-538 – ISSN 1611-2156
Received: December 17, 2024, accepted: February 25, 2025, published: April 01, 2025

Supplementary Table 1: Additional Predicted ADMET properties

Compound	Caco2 permeability (> 0.90)	No. Rotatable Bonds (> 10)	SASA (300.0–1000.0)	S+MDCK (5-1130)
**Sulfadiazine**	0.36	4	475.42	167.49
**FQ5**	0.18	6	598.54	7.78
**FQ6**	1.31	3	519.07	249.23
**FQ7**	1.04	4	513.25	791.88
**FQ12**	0.18	6	595.53	109.41

Abbreviations: Caco2 permeability = permeability of compound across a human colon carcinoma cell line monolayer; SASA = solvent accessible surface area; S+MDCK = solubility plus Madin-Darby canine kidney (MDCK) cell permeability
